# High carrier frequency of a nonsense p.Trp230* variant in *HSD3B2* gene in Ossetians

**DOI:** 10.3389/fendo.2023.1146768

**Published:** 2023-05-16

**Authors:** Nina Makretskaya, Natalia Kalinchenko, Inna Tebieva, Sofya Ionova, Rena Zinchenko, Andrey Marakhonov, Anatoly Tiulpakov

**Affiliations:** ^1^ Department of Genetics of Endocrine Diseases, Research Centre for Medical Genetics, Moscow, Russia; ^2^ Institute of Pediatric Endocrinology, Endocrinology Research Centre, Moscow, Russia; ^3^ Consulting and Diagnostic Department, Republic of North Ossetia-Alania (RNOA) “Republican Children’s Clinical Hospital”, Vladikavkaz, Russia

**Keywords:** CAH, real-time PCR, HSD3B2, deficiency of 3β-hydroxysteroid dehydrogenase, Ossetians

## Abstract

**Background:**

Congenital adrenal hyperplasia (CAH) caused by 3β-HSD deficiency is a rare form of congenital adrenal deficiency with an autosomal recessive type of inheritance. Previously we have demonstrated that a single nucleotide variant (SNV) p.Trp230* in the homozygous state is a frequent cause of CAH among the indigenous population of North Ossetia-Alania represented by Ossetians.

**Methods:**

Genotyping of the NM_000198.3:c.690G>A p.Trp230* variant was performed by Real-time PCR. 339 healthy individuals of Ossetian origin were included in the study. Allele frequencies, Fisher’s confidence intervals (CI) were calculated using the WinPepi v. 11.65 software. Comparison of allele frequencies was performed with the z-score test for two proportions.

**Results:**

Eight heterozygous carriers of c.690G>A variant in HSD3B2 gene were detected in 339 samples investigated. The total allele frequency of p.Trp230* variant was 0.0118 (n=8/678, 95% CI=0.0051–0.0231). Accordingly, the heterozygous carrier rate was 0.0236 (n=8/339). The frequency of CAH caused by p.Trp230* variant in HSD3B2 in Ossetian population was 1:7183 or 13.9 per 100,000 (95% CI: 1:1874–1:38447 or 3–53 per 100,000).

**Conclusion:**

The results demonstrate high frequency of p.Trp230* variant in Ossetians, which is most likely attributed to a founder effect.

## 1 Introduction

Congenital adrenal hyperplasia (CAH) is a group of autosomal recessive disorders caused by impairment at one of the steps of cortisol biosynthesis in adrenal cortex. To date, 7 distinct clinical forms of CAH and the corresponding genes are recognized, namely: lipoid adrenal hyperplasia, *STAR* (OMIM #201710); deficiency of cholesterol side-chain cleavage enzyme, *CYP11A1* (OMIM #613743); deficiency of 3β-hydroxysteroid dehydrogenase, *HSD3B2* (OMIM #201810); deficiency of 17α-hydroxylase/17,20-lyase, *CYP17A1* (OMIM #202110); deficiency of P450 oxidoreductase, *POR* (OMIM #613571); deficiency of 21-hydroxylase, *CYP21A2* (OMIM #201910); and deficiency of 11β-hydroxylase, *CYP11B1* (OMIM #202010) (1). The two most frequent enzymatic defects, deficiencies of 21-hydroxylase and 11β-hydroxylase, account for 90-99% (2, 3) and 5-8% (4) of all CAH cases, respectively, whereas the other 5 forms are exceptionally rare.

*HSD3B2* is expressed in adrenal cortex and gonads where it converts Δ5-3β-hydroxysteroids into the corresponding Δ4-3-keto isomers: namely, pregnenolone to progesterone, 17α-hydroxypregnenolone (17OHPreg) to 17α-hydroxyprogesterone (17OHP) and dehydroepiandrosterone (DHEA) to androstenedione, respectively. Biallelic loss-of-function variants in *HSD3B2* present with salt wasting, genital ambiguity, and hypogonadism in both sexes (5). The disorder is estimated to have an overall incidence of less than 1/1 000 000 live births (6), however the prevalence may be substantially higher in inbred populations (7).

Previously we have reported 2 siblings with 3β-hydroxysteroid dehydrogenase (3β-HSD) deficiency caused by homozygous p.Trp230* variant in *HSD3B2* (8). Subsequently additional patients with the same variant have been described, all of which, like the first two, were of Ossetian origin (9). Based on the number of the diagnosed cases in North Ossetia–Alania (RNOA) in 2007–2017 the incidence of 3β-HSD deficiency was estimated to be 1:22470, close to that calculated during the same period for 21-hydroxylase deficiency (1:18750) (9). The above findings prompted us to study carrier frequency of the p.Trp230* variant in Ossetians.

There are 5 subethnic groups within Ossetian origin: Ironians, Digorians, Kudar, Iasi, Dvals. In this study, a comparative analysis was carried out of two of them: Ironians and Digorians.

## 2 Method

### 2.1 Ethical consideration

A written informed consent for genetic studies was obtained from the subjects. The study was conducted according to the guidelines of the Declaration of Helsinki and approved by the Institutional Review Board of the Research Centre for Medical Genetics, Moscow, Russia (protocol no. 7 of December 20, 2017).

### 2.2 Variant nomenclature, accession, and pathogenic status

GenBank entry NM_000198.3 was used as reference for the *HSD3B2* gene nucleotide and amino acid numbering. All genome coordinates are given according to the GRCh37/hg19 assembly. Assessment of pathogenic status of the variant was performed using ACMG/AMP recommendations (10). Prediction whether variant is a subject to degradation by nonsense-mediated decay (NMD) was made using NMDEscPredictor (11).

### 2.3 Genetic testing and autozygosity mapping

DNA samples were analyzed using chromosomal microarray analysis (CMA). The analysis was performed using the GeneChip™ 3000 system (Thermo Fisher Scientific, USA) according to the manufacturer’s protocol on a CytoScan HD microarray containing 2.67 million markers.

Regions of autozygosity were analyzed using Chromosome Analysis Suite v. 4.2.1 software (Thermo Fisher Scientific, USA) with the subsequent visual inspection of the regions’ borders. The age of the mutation event was estimated using the method described in detail elsewhere (12). For the interpolation of hg19 physical positions into the sex-averaged map positions in Kosambi cM, Rutgers Maps v.3 were used (13).

Allele frequencies, Fisher’s confidence intervals (CI) were calculated using the WinPepi v. 11.65 software (14). Comparison of allele frequencies was performed with the *z*-score test for two proportions.

### 2.4 Subjects

The study included 339 healthy Ossetians, with a distribution by subethnic groups: 283 Ironians and 56 Digorians. Sex ratio is 32.5:67.5%, median age is 37, interquartile range 26–52 years, range 3–86.

### 2.5 Real-time PCR

Genomic DNA was isolated from peripheral blood leukocytes using the PureLink^®^ Genomic DNA Mini Kit (Thermo Scientific, Waltham, MA, USA) according to the method recommended by the manufacturer.

Genotyping of the nucleotide variant NM_000198.3:c.690G>A (p.Trp230*) was performed by real-time PCR on a StepOne Plus amplifier (Thermo Scientific, Waltham, MA, USA) using the following primers and TaqMan probes:

HSD_4F CCTTGTACACTTGTGCGTTAAGACCCACA,

HSD_4R CCATCTGGAATCAAGGCGGAGGTCA,

HSD_wt (VIC)-CGGCCTGGGCCCACATTCTGGCCG-(BHQ2),

HSD_mut (FAM)-CGGCCTGAGCCCACATTCTGGCCG-(BHQ1).

## 3 Results

Previously, it was shown that a single nucleotide variant NM_000198.3:c.690G>A (chr1:119964814G>A, GRCh37/hg19), which produces a premature translation termination codon at position 230 of HSD3B2 protein (p.Trp230*) in the homozygous state is the major causes of CAH in the indigenous population of RNOA represented by Ossetians (9).

In order to determine the population frequency of the identified causative variant in *HSD3B2* gene the population study was performed.

Among the 339 samples investigated, the variant c.690G>A was detected in 8 cases of heterozygous carriage. In Ironians, this substitution was detected in 7 cases, with allele frequency of 0.0124 (n=7/566, 95% CI=0.0036–0.0300). In the Digorian subethnic group, the variant was found in 1 case, the frequency is 0.0089 (n=1/112, 95% CI=0.0002–0.0487). There was no statistically significant difference in the allelic frequency of c.690G>A variant between the subpopulations of Ironians and Digorians (*z*-score = 0.3079, p = 0.7566), which indicates a single origin of the variant before the division of the Ossetian people into sub-ethnic groups and allows us to combine the data to determine the overall frequency of the disease.

The total allelic frequency of the studied variant is 0.0118 (n=8/678, 95% CI=0.0051–0.0231). Accordingly, the heterozygous carrier rate is 0.0236 (n=8/339). Based on the Hardy-Weinberg equilibrium, the proportion of homozygotes for the mutant allele is 0.000139, which gives the prevalence of the disease 1:7183 subjects or 13.9 subjects per 100,000 population (95% CI=1:1874–1:38447 or 3–53 subjects per 100,000 population). The frequency of heterozygous carriers of c.690G>A variant is 1:43 subjects (95% CI: 1:22–1:99).

Since the proportion of closely related marriages among Ossetians is extremely low (15) such high estimated frequency of 3β-HSD deficiency in the studied population suggests a founder effect in the distribution of the c.690G>A variant in the indigenous population.

**Figure 1:**
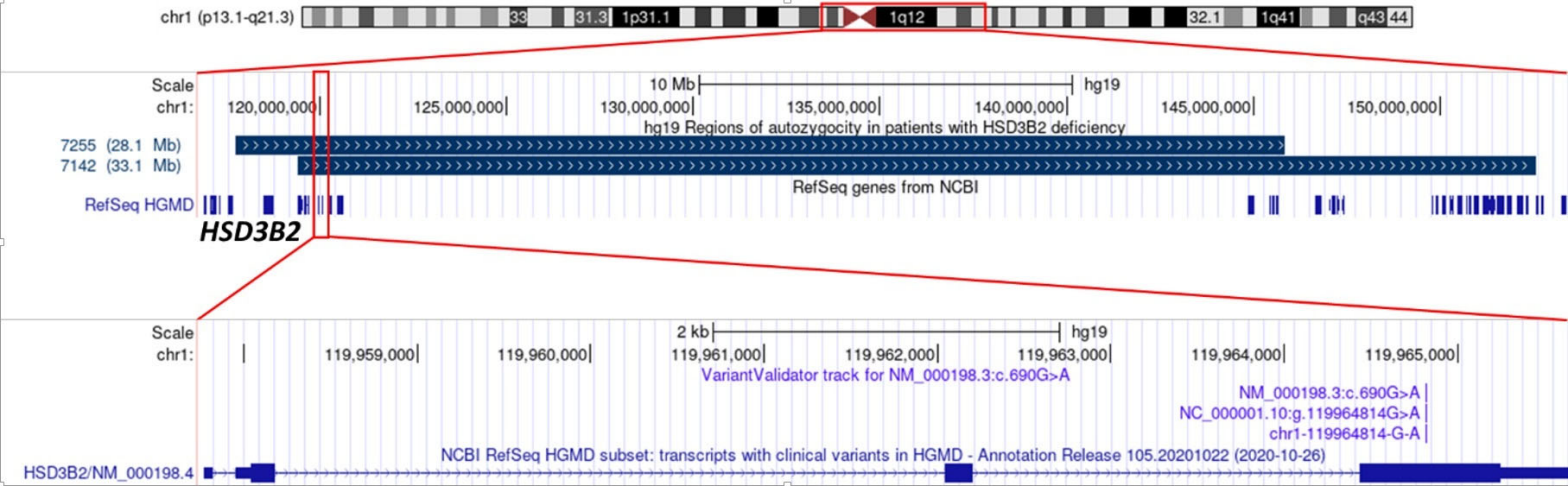
The position of extended autozygosity regions on chromosome 1 (upper panel), including the locus of the *HSD3B2* gene (middle panel); variant c.690G>A p.Trp230* in exon 4 (lower panel).

To test this hypothesis we performed a haplotype study using genotyping with Affymetrix high-density SNP Array in two patients with homozygous c.690G>A variant. As a result, we detected autozygosity regions at chr1:119425396_152538358 and chr1:117767918_145829474 encompassing the HSD3B2 locus and overlapping the centromere of the chromosome 1 (shown in **Figure 1**).

Using Rutgers Map v.2 (13) the physical boundaries of autozygosity regions determined by the SNP array were converted into 6.06 and 3.44 cM (Kosambi) sex-averaged genetic distances, which corresponds to a common ancestor of 8.25–14.53 generations ago. Based on the average length of generations in humans of 25 years (12), these estimates suggest the age of the mutation of 206.21–363.32 years.

## 4 Discussion

To our knowledge, apart from the observations in Amish (7), the previously reported cases of Ossetian origin (8, 9) represent the largest cohort of patients with 3β-HSD deficiency caused by the same variant.

The variant NM_000198.3:c.690G>A p.Trp230* (chr1:119964814G>A) is not present in gnomAD (16) and Human Gene Mutation Database (HGMD) (www.hgmd.cf.ac.uk). Nevertheless, in addition to our cases (8, 9) the variant was described in a compound-heterozygote state (p.[Ala82Asp];[Trp230*]) in a patient “of Russian origin” reported by Nordenström et al. (17). It cannot be ruled out that that heterozygous variant has the same origin as the cases detected by our group.

Analysis of the probable molecular consequences of the detected p.Trp230* variant suggests that it most likely does not activate the mechanism of nonsense-mediated mRNA decay according to the NMDEscPredictor (11), since it is located in the last exon (shown in **Figure 1**), but leads to the synthesis of a shortened by 143 (out of 372) amino acid residues of HSD3B2 protein. The lost residues include a 21-amino acid sequence (positions 287-307) that is essential for fixation of the enzyme at the inner surfaces of the membranes of cellular organelles (18). According to the ACMG criteria, the variant can be classified as pathogenic with a significance level of PM2, PVS1, PP1-M (10).

We have demonstrated that the estimated prevalence of 3β-HSD deficiency due to this founder mutation (1:7183) is higher than the prevalence of the most common form of CAH, 21-hydroxylase deficiency, in the majority of studied populations (1). To date, the sensitivity of 17-OHP-based neonatal screening for 3β-HSD deficiency is not known. Taking into account that in the simple virilizing and nonclassic forms of 21OH-deficiency the sensitivity may be as low 79.7% and 32.4%, respectively (19), missed cases of 3β-HSD deficiency cannot be ruled out. The regional screening data of 2007–2017 showed the incidence that was lower than theoretically expected (1:22470 vs 1:7183), although the difference was not significant (*z*-score = 0.8509, p = 0.3953). In the literature, several reports document detection of 3β-HSD deficiency by the 17-OHP-based neonatal screening (9, 20, 21), however both negative (20) and borderline (21) results also exist. Further studies in North Ossetia-Alania will show whether modifications for the national screening program are needed to improve the diagnostics of 3β-HSD deficiency for this region. Direct steroid hormone quantification in dried blood spots using ultra-performance liquid chromatography-tandem mass spectrometry (LC-MS/MS) (22) or a lower 17-OHP immunoassay cut-off combined with LC-MS/MS as the second-tier test (23, 24) may be considered as the options.

The high estimated allele frequency of the p.Trp230* variant in *HSD3B2* gene as well as identification of extended regions of autozigosity in two unrelated patients with 3β-HSD deficiency of Ossetian origin could suggest the possible “founder effect” as well as the “bottleneck” effect in Ossetians. These observations are supported by the fact that before the 1930s, most of the population of Russia lived in rural areas, practicing assorted marriages based on geographical proximity and ethnicity. In addition, at the end of XVIII – early XIX centuries, the population of the RNOA decreased 10 times (from 200,000 people to 20,000) due to the spread of plague in this area, which also corresponds to the identified age of the mutation (25).

## Data availability statement

The datasets presented in this study can be found in online repositories. The names of the repository/repositories and accession number(s) can be found in the article/supplementary material.

## Ethics statement

The studies involving human participants were reviewed and approved by the Institutional Review Board of the Research Centre for Medical Genetics, Moscow, Russia (protocol no. 7 of December 20, 2017). The patients/participants provided their written informed consent to participate in this study.

## Author contributions

NM: application of statistical study data, conducting a research and investigation process, specifically performing the experiments and data collection; preparation, creation and presentation of the published work; acquisition of the financial support for the project leading to this publication. NK: conducting a research and investigation process, specifically performing the experiments and data collection, provision of study materials. IT: conducting a research and investigation process, specifically performing the experiments and data collection, provision of study materials. SI: Application of statistical study data; conducting a research and investigation process, specifically performing the experiments and data collection. RZ: formulation or evolution of overarching research goals and aims, provision of study materials; preparation, creation and presentation of the published work; management and coordination responsibility for the research activity planning and execution. AM: application of statistical study data, conducting a research and investigation process, specifically performing the experiments and data collection; preparation, creation and presentation of the published work. AT: formulation or evolution of overarching research goals and aims, development or design of methodology; preparation, creation and presentation of the published work, management and coordination responsibility for the research activity planning and execution. All authors contributed to the article and approved the submitted version.
